# Serious Games for Improving Technical Skills in Medicine: Scoping Review

**DOI:** 10.2196/24093

**Published:** 2021-01-25

**Authors:** Tycho Joan Olgers, Anne Akke bij de Weg, Jan Cornelis ter Maaten

**Affiliations:** 1 Department of Internal Medicine University of Groningen University Medical Center Groningen Groningen Netherlands

**Keywords:** serious games, technical skills, ultrasound skills, validity of serious games

## Abstract

**Background:**

Serious games are being used to train specific technical skills in medicine, and most research has been done for surgical skills. It is not known if these games improve technical skills in real life as most games have not been completely validated.

**Objective:**

This scoping review aimed to evaluate the current use of serious games for improving technical skills in medicine and to determine their current validation state using a validation framework specifically designed for serious games.

**Methods:**

We used the Preferred Reporting Items for Systematic Reviews and Meta-Analyses extension for Scoping Reviews (PRISMA-ScR) guidelines. A multidatabase search strategy was adopted, after which a total of 17 publications were included in this review.

**Results:**

These 17 publications described five different serious games for improving technical skills. We discuss these games in detail and report about their current validation status. Only one game was almost fully validated. We also discuss the different frameworks that can be used for validation of serious games.

**Conclusions:**

Serious games are not extensively used for improving technical skills in medicine, although they may represent an attractive alternative way of learning. The validation of these games is mostly incomplete. Additionally, several frameworks for validation exist, but it is unknown which one is the best. This review may assist game developers or educators in validating serious games.

## Introduction

Point-of-care ultrasound is an important bedside diagnostic tool for various specialties. For internal medicine, it is a relatively new tool, and educational programs have been created in The Netherlands for residents and internists to become competent in ultrasound [1]. Learning to make the right probe movements and constructing a 3D mental image from a 2D screen image may cost time. To assist in training eye-hand coordination with an ultrasound probe, a serious game involving a 3D-printed probe and an underwater game is under development in The Netherlands [2]. However, it is not known if this game will actually improve ultrasound skills (technical skills of probe movements and thereby image optimization) in real practice. To the best of our knowledge, there is no serious game available at this moment for learning ultrasound skills. A review in 2012 showed that some games were available to train other technical skills like laparoscopic psychomotor skills, but none of the serious games had completed a full validation process [3]. In this review, we aimed to explore the current use of serious games for training technical skills in medicine, including personal factors of influence while playing these games, and we determined their validation status using a framework for assessing the validity of serious games [4,5]. Knowledge about validation and the current use of serious games for technical skills may provide useful information to develop games for training ultrasound skills.

We have determined the following research questions: (1) Which games exist for training technical skills in medical education or practice? (2) What is known about the validity of these games? (3) Which personal factors influence the performance in these games?

## Methods

### Identification of Relevant Studies

We conducted a scoping review using the recommended items from the Preferred Reporting Items for Systematic Reviews and Meta-Analyses extensions for Scoping Reviews (PRISMA-ScR) guidelines [6]. We included original studies investigating serious games for technical skills in health care. Studies were excluded if they (1) evaluated nontechnical skills, such as cognitive skills; (2) included a game that was designed as a therapy for patients or to teach anything other than a medical intervention; (3) did not have full text available; (4) were written in a non-English language; and (5) only described a simulator instead of a serious game.

**Figure 1:**
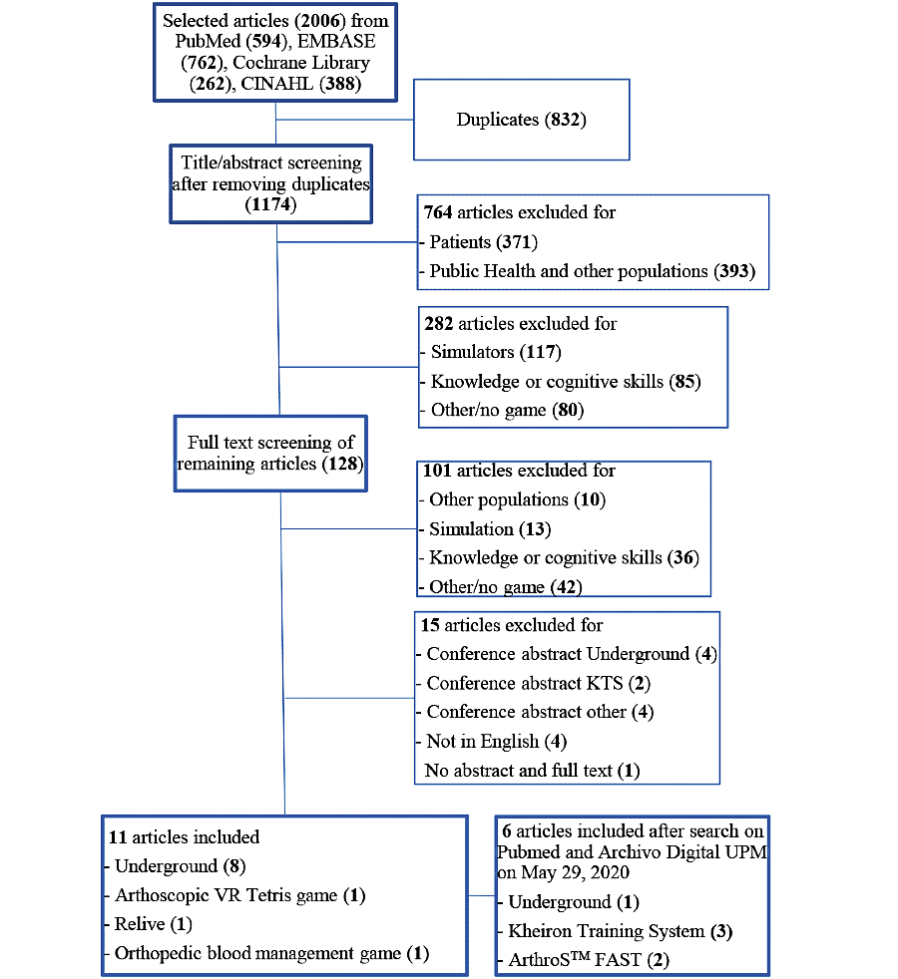
Flow chart of study inclusion. KTS: Kheiron training system.

The databases PubMed, Cochrane Library, EMBASE, and CINAHL were searched on April 9, 2020, using the following terms or abstracts of these terms without limitation of published date: serious game, video game, computer game, education, teaching, training, and skill. This search resulted in 2006 articles (Figure 1). One Author (AbdW) screened all articles and removed duplicates (n=832). After reading the title and/or abstract, another 764 articles were excluded, as they did not concern medical skills, and 282 were excluded for other previously defined reasons (only simulators, describing only cognitive skills, or no game at all). The inclusion and exclusion criteria are presented in Textbox 1. From the 128 remaining articles, the full text was obtained, after which another 101 were excluded for the previously mentioned reasons that could not be determined by the title/abstract. Additionally, we excluded one article because the abstract and full text were not available, four articles because they had non-English text, and 10 articles because they concerned only conference abstracts. The remaining 11 articles were critically assessed, after which six additional articles were found that had not been included in our original search (May 29, 2020); one article was published after our query, and two additional articles were found with a specific google search for “arthroscopy VR Tetris game.” The article describing a game for arthroscopy, included in our primary search, refers to it as the “arthroscopy VR Tetris game.” A specific search on Google for this term and VirtaMed, the operating platform, revealed that this game appears to be part of the ArthroS FAST simulator. A search on PubMed for “ArthroS FAST” and “arthroscopy” produced one article, which, in turn, cited another relevant article about this simulator. However, it was described as a simulator and not as a game, and therefore, was not found in the primary search. Finally, three articles were only found with a specific google search based on two conference abstracts related to the primary search. Full text was not published but could be found on the internet separately. Our strategy described above identified 17 articles to be included in this review.

**Inclusion and exclusion criteria.**

**Inclusion criteria**

- The game was designed to teach a medical intervention usually performed by health care personnel.

- The game was designed to teach a technical skill.

- The technical skill was performed by the player and simulated the real-life technical skill.

- The game was described as a serious game in at least one article.

- The article was available in full text and in the English language.

**Exclusion criteria**

- The game was designed as a therapy for patients or to teach anything other than a medical intervention.

- The game was designed to teach nontechnical skills (not involving hands-on activity), for example, only knowledge, cognitive skills, and attitudes.

- The technical skills used in the game do not resemble real-life technical skills (training of cognitive skills).

- The approach was described solely as a simulator or other medium (eg, courses, online modules, and mannequin).

- The conference abstract or article was available in a non-English language.

### Validity Types for Assessment of Games

Validity in game design for technical skills means that playing the game will actually improve the specific skill in real life. There are several frameworks used for assessment of game validity. The classical framework consists of five different types of validity, and more integrative models are exploring different sources of validity [7]. However, as most reported studies on game validity for technical skills use the classical framework, we have chosen to use that framework for this review. This classical framework consists of the following five different phases of validity: content validity, face validity, construct validity, concurrent validity, and predictive validity [4,5]. Content validity concerns the content of the game to be legitimate (eg, its specifications: Is the game complete and correct, and has nothing but the intended construct [no additional content other than what it was designed for]?). Face validity means that the game appears to be similar to the construct it attempts to represent and is essentially the concept of the game (Do educators or trainees view it as a valid way of instruction?). Construct validity means the game actually measures (or trains) what it intends to measure (Is the game able to measure different skills?). It can be determined by testing prototypes and comparing scores of experts in real life to those of novices. The last stage integrates the construction phase with performance in real life. Concurrent validity reflects the correlation between the performance in the serious game and the performance with the actual instrument. Predictive validity relates the performance in the game to outcomes in reality or predicts skills in real life. In theory, this may require a randomized controlled trial. If the type of validity was not explicitly mentioned, we interpreted the experiment that was conducted and scored the applicable validity.

## Results

### Search Strategy

The abovementioned search strategy resulted in 17 articles describing five different serious games to train technical skills in health care. We will discuss these five games and their current validity state.

### Underground Game

The “Underground” game is the most extensively studied and described. A total of nine articles discussed this Nintendo Wii-U–based game for training basic laparoscopic skills [8-16]. The game was released in 2015 and uses two Wii remote controllers in a custom-made laparoscopic tool shell. The aim is to save robots in a fictional mining world by demolishing and rebuilding the environment. The learning objectives include learning inverted movements, eye-hand coordination, depth perception, and ambidexterity. The face validity, construct validity, and concurrent validity have already been demonstrated and published. It has been shown that playing the game in advance to laparoscopic simulated tasks increased skills [9,16]. Additionally, a study using the game as a preoperative warm-up for 15 minutes showed improvement in task performance [12]. However, the final stage of predictive validity has not been completed yet. This may require comparing surgical skills in the operating theatre between surgeons playing and those not playing the game before the surgery. Several participant characteristics were assessed during these studies. Men outperformed women, and prior video game experience was correlated with “Underground” game scores, although independence of these two variables could not be established as women had less video game experience [10].

### Arthroscopic VR Tetris Game

The setup integrates the well-known game “Tetris” into a virtual reality platform for arthroscopic training. The platform consists of a dome with several entry portals for the camera and graspers, and a video screen. The participant can manipulate the Tetris blocks to the preferred position before putting them down. As in the real Tetris game, a line is cleared if it is completely filled with blocks. The aim is to clear 10 lines. The learning objective in this game is to train motor skills, such as opening and closing of graspers and eye-hand coordination. A construct validity study used this setup but with a different assignment, consisting of three activities, and compared the following three groups of users: postgraduate students, fellows, and faculty [17]. Strikingly, the combined scores of the three activities did correlate with year of training but not with prior total arthroscopies performed. It would be expected that a higher year of training relates to a higher number of arthroscopies performed and therefore higher scores. Unfortunately, an explanation of this finding was not provided. It is possible that these associations were not significant because the sample size was small or because the game design itself was unable to discriminate the three groups. It is important to emphasize that two validation studies used the arthroscopic simulator setup but not the serious game Tetris. This means that the serious game itself was not validated and that the setup was in fact an arthroscopy simulator. One study with the Tetris game showed that residents performed better with their dominant hand, but this difference disappeared in experienced surgeons [18]. Unfortunately, scores between residents and surgeons were not compared. Interestingly, the second study using the arthroscopic setup showed a gender difference in performance unrelated to previous experience [19].

### Kheiron Training System

The Kheiron training system is a serious game for minimally invasive surgery training. The setup includes a box trainer with a box and a camera inside it, real laparoscopic instruments (in contrast to the game “Underwater” that uses the Wii console), a computer, and a monitor. The game is about a young alchemist who has to find the Philosopher’s stone by completing different recipes. Two articles provided technical details of the setup and machine learning, but without any kind of validation of the game itself. Only one additional article described the start of the content validity of this game [20]. Other validation studies were announced but have not been published yet.

### Relive Game

The setup of Relive consists of a motion detection device (Kinect version 1; Microsoft Corp), a Resusci Anne mannequin, and a laptop. The game is staged on the planet Mars where chest compressions have to be performed on a person with real-time feedback. It can be played in tournament mode. The game was evaluated by a small study with 65 students who played the game at three different time intervals [21]. After a few months, chest compression depths were better than at baseline. However, there was no comparison with students who did not play the game, and no validation was performed in terms of concurrent and predictive validity.

### Orthopedic Blood Management Game

This game consists of a computer, a screen, and a haptic device to manipulate surgical instruments and has been developed to train eye-hand coordination by manipulating instruments to stop bleeding on surfaces and in a virtual patient. The game was tested with students, and a subsequent questionnaire indicated that they found the game realistic and helpful, which is the first step in determining content and face validity [22]. Other validation studies have not been published.

An overview of the included articles, the games they discuss, and the validity types is provided in Table 1 [8-24].

**Table 1:** Articles included in the review.

Authors	Article title	Game	Validity
Jalink et al [8]	Construct and concurrent validity of a Nintendo Wii video game made for training basic laparoscopic skills	Underground	Construct and concurrent validity
IJgosse et al [9]	Saving robots improves laparoscopic performance: transfer of skills from a serious game to a virtual reality simulator	Underground	Concurrent validity
IJgosse et al [10]	Construct validity of a serious game for laparoscopic skills training: validation study	Underground	Construct validity
Goris et al [11]	Training basic laparoscopic skills using a custom-made video game	Underground	Concurrent validity
Jalink et al [12]	The effect of a preoperative warm-up with a custom-made Nintendo video game on the performance of laparoscopic surgeons	Underground	Concurrent validity
Jalink et al [13]	Face validity of a Wii U video game for training basic laparoscopic skills	Underground	Face validity
Rosser Jr et al [14]	Impact of Super Monkey Ball and Underground video games on basic and advanced laparoscopic skill training	Underground	Concurrent validity
Overtoom et al [15]	Training in basic laparoscopic surgical skills: residents’ opinion of the new Nintendo Wii-U laparoscopic simulator	Underground	Construct and face validity
Harrington et al [16]	Playing to your skills: a randomised controlled trial evaluating a dedicated video game for minimally invasive surgery	Underground	Construct and concurrent validity
Tofte et al [17]	Knee, shoulder, and fundamentals of arthroscopic surgery training: validation of a virtual arthroscopy simulator	Arthroscopic VR (Tetris) game	Construct validity
Pedowitz et al [18]	Asymmetry in dominant/non-dominant hand performance differentiates novices from experts on an arthroscopy virtual reality serious game	Arthroscopic VR (Tetris) game	Construct validity
Walbron et al [19]	Evaluation of arthroscopic skills with a virtual reality simulator in first-year orthopaedic residents	Arthroscopic VR (Tetris) game	Construct validity
Sanchez Peralta et al [20]	E-learning serious game for surgical skills training: Kheiron training system	Kheiron Training System	Content validity
Sanchez Peralta et al [23]	Serious game for psychomotor skills training in minimally invasive surgery: Kheiron Training System	Kheiron Training System	Only technical description of the setup, no validation of the game
Martin Vicario et al [24]	Automatic detection of surgical instruments’ state in laparoscopic video images using neural networks	Kheiron Training System	Only description of machine learning of the instrument state, no validation of the game
Semeraro et al [21]	Kids (learn how to) save lives in the school with the serious game Relive	Relive	Construct validity
Qin et al [22]	Learning blood management in orthopedic surgery through gameplay	Orthopedic blood management	Content and face validity

## Discussion

### Principal Results

This review provides an overview of the currently used serious games for improving technical skills in health care and the subsequent validation process. We reviewed 17 articles describing five serious games available for improving technical skills. The game “Underground” has been the most extensively validated, including content, face, construct, and concurrent validity. Most other games only had a description of the initial steps in the process of validation. This means that we are not sure if playing the game will lead to better performance of that specific skill in clinical practice. Most serious games for technical skills need additional validation studies. Guidelines on how to perform validation for serious games may assist game designers and educational experts to develop these games. It is necessary for serious games to be well constructed and evaluated and to impact trainees’ performance in real life, especially if expensive software or equipment is needed to play the game.

### Frameworks for Validation

Validation of serious games is the process of collecting and interpreting validity evidence, which, in this case, is used to evaluate the appropriateness of a game for improving technical skills in real life [25]. The classical validation framework identified at least three different “types” of validity (content, construct, and criterion). Criterion validity includes correlational, concurrent, and predictive validity and denotes the correlation between actual test scores and the “true” (criterion) scores, for example, the correlation with a gold standard. The specifically designed framework for serious games, suggested by Graafland and Warmelink, also uses content and construct validity but adds concurrent and predictive validity instead of criterion validity [4,5]. A more contemporary framework was proposed in 1989 and finally adopted as standard in this field in 1999 and 2014 [26]. Many elements of the classical validation framework are recognizable in this framework, including the construction of the game and its effect on task performance in real practice. It consists of the following five sources of evidence: content, internal structure, relationship with other variables, response process, and consequences [14,15]. Finally, the most recent validation framework was proposed by Kane [27]. The model of Kane is based on inferences and consists of scoring, generalization, extrapolation, and implication. If we apply this framework to serious games, it would start with a player who has a specific score (performance of technical skills) in the game. We assume that the score reflects the overall level of performance, but this score is very dependent on the scoring system/game itself. Multiple scores (or game levels) are combined to generate a total score, assuming this better reflects the performance (technical skills in our case) across the whole test domain (internal consistency). The generalization of the score still deals with performance in the test world and reflects how well the selected test items (scores) represent all of the theoretically possible items. Next, this test world performance is extrapolated to the real world, assuming that this test performance also reflects the skills in real life. Evidence to support extrapolation can be collected by comparing test results with a conceptually related real-world assessment. The final stage is the impact/consequence of this assessment (eg, performance in the game) on the real world (eg, clinical performance, patient safety, length of training, and pass/fail standard). Important questions are as follows: Will playing the game improve or predict technical skills in real life and what are the potential consequences for the trainee?

Although different frameworks for serious games may be used, the validation has the following two key elements: evidence must be collected about the construct of the game itself and its effects on performance in real life. The type of evidence may vary across different games and stages of validation.

### Serious Game, Gamification, and Simulation

There is considerable overlap between a serious game, gamification, and a simulator. A serious game is an interactive computer application, with or without specific hardware, that has a challenging goal, is engaging, incorporates some scoring system, and increases the skills, knowledge, or attitudes of its user [3]. These games are designed for specific objectives and therefore differ from commercial video games. A serious game differs from a simulator or gamification in that it uses another context than the actual performance in real life. The game “Underground” is an example of a serious game. In this game, no surgical task is performed or simulated, but the goal is to improve surgical skills. In gamification, there is addition of a game or gaming elements to a nongame context. The game “Relive” is an example of gamification. It uses a normal mannequin to train chest compression but with a scoring element, and the mannequin is “lying on Mars.” A simulator is a device that enables the operator to reproduce or represent under test conditions those phenomena that are likely to occur in actual performance. In health care, simulators can be high fidelity, which means they have a high resemblance to the actual context, for example, a fully equipped operating theatre with a mannequin as a patient instead of a real patient. In this review, the “Arthroscopic VR Tetris game” is actually a simulator with realistic instruments, but instead of a virtual patient, it uses the game Tetris, although the same setup is also used for more realistic simulations.

We have attempted to identify factors that influence performance within a game. Unfortunately, the results were inconsistent and differed between games and simulators. It seems that previous gaming experience is at least an independent predictor of game performance. It is unknown if there is also gender inequality. These are interesting topics for future research.

The idea for this review originated from our interest in multiple learning modalities for learning ultrasound skills. However, there are no specific serious games for learning ultrasound skills available at this moment. It is noteworthy that one article describes a serious game for ultrasound-guided needle placement [28]. Although the authors use an interesting setup for learning needle placement, we excluded this article from our review, because in our opinion, it is an ultrasound simulation setup with some gaming elements (gamification) but not a serious game.

### Limitations

The literature selection process was primarily done by one reviewer, which may have caused selection bias. However, we used stringent criteria formulated in advance of our search and we received assistance from our experienced librarian. Additionally, the first author cross-checked PubMed for missed publications, although an extensive second search was not performed. Nevertheless, we are confident that no relevant publications have been missed. The search strategy included the word “skill” to eliminate serious games concerning cognitive skills. However, relevant articles describing serious games for technical skills using different words for skills may have been missed, although in the references, we did not find any relevant articles without the word “skill.” Moreover, an additional search on PubMed with “psychomotor skills” or “psychomotor performance” did not result in additional articles. It is possible that serious games are being used for teaching technical skills but without any publication in the medical literature. We were not able to address the specific quality of each study as there are no specific quality criteria for validation studies of serious games. We excluded four non-English articles and 10 conference abstracts. We translated and reviewed them, but they were not of additional value. Thus, the exclusion did not cause relevant selection bias.

### Conclusions

To date, only a few serious games exist for the training of technical skills in medical education, and serious games for learning ultrasound skills are lacking. Factors predicting performance in serious games are only briefly known. The majority of games still need full validation. This is especially true if they require expensive software and/or hardware. Serious games can be evaluated with the classical concept of validation consisting of content validity, face validity, construct validity, concurrent validity, and predictive validity, although more integrative frameworks are advocated. This review may help serious game developers in the validation process of their games. Despite the specific process of validation, the ultimate goal of serious games is to improve technical skills in real life in a fun way.
